# Adolescent Running Biomechanics - Implications for Injury Prevention and Rehabilitation

**DOI:** 10.3389/fspor.2021.689846

**Published:** 2021-08-26

**Authors:** Simon C. McSweeney, Karin Grävare Silbernagel, Allison H. Gruber, Bryan C. Heiderscheit, Brian J. Krabak, Mitchell J. Rauh, Adam S. Tenforde, Scott C. Wearing, Astrid Zech, Karsten Hollander

**Affiliations:** ^1^School of Clinical Sciences, Faculty of Health, Queensland University of Technology, Brisbane, QLD, Australia; ^2^Department of Physical Therapy University of Delaware, Newark, NJ, United States; ^3^Department of Kinesiology, School of Public Health – Bloomington, Indiana University, Bloomington, IN, United States; ^4^Department of Orthopedics and Rehabilitation, University of Wisconsin, Madison, WI, United States; ^5^Department of Rehabilitation, Orthopedics and Sports Medicine, University of Washington and Seattle Childrens Hospital, Seattle, WA, United States; ^6^Doctor of Physical Therapy Program, San Diego State University, San Diego, CA, United States; ^7^Department of Physical Medicine and Rehabilitation, Harvard Medical School, Spaulding Rehabilitation Hospital, Boston, MA, United States; ^8^Department of Human Movement Science and Exercise Physiology, Institute of Sport Science, Friedrich Schiller University Jena, Jena, Germany; ^9^Institute of Interdisciplinary Exercise Science and Sports Medicine, Faculty of Medicine, MSH Medical School Hamburg, Hamburg, Germany

**Keywords:** biomechanical, youth, running-related injuries, kinetics, kinematics, footstrike pattern

## Abstract

Global participation in running continues to increase, especially amongst adolescents. Consequently, the number of running-related injuries (RRI) in adolescents is rising. Emerging evidence now suggests that overuse type injuries involving growing bone (e.g., bone stress injuries) and soft tissues (e.g., tendinopathies) predominate in adolescents that participate in running-related sports. Associations between running biomechanics and overuse injuries have been widely studied in adults, however, relatively little research has comparatively targeted running biomechanics in adolescents. Moreover, available literature on injury prevention and rehabilitation for adolescent runners is limited, and there is a tendency to generalize adult literature to adolescent populations despite pertinent considerations regarding growth-related changes unique to these athletes. This perspective article provides commentary and expert opinion surrounding the state of knowledge and future directions for research in adolescent running biomechanics, injury prevention and supplemental training.

## Introduction

Youth running participation is increasing throughout the world, with global participation rates of adolescents reported among the top three sport activities in most regions (Hulteen et al., 2017). Adolescents are defined by the World Health Organization as individuals aged 10–19 years (World Health Organization, 2018). However, the “biological” age or maturational development of adolescent athletes may be of greater significance with respect to running-related injuries (RRI). Changes in tissues such as bone, tendon, muscle, cartilage and growth plate occur at varied rates and locations during times of rapid growth (Lloyd et al., 2014; Krabak et al., 2016a). Maturation of these tissues during puberty is affected by hormonal, genetic and environmental factors which may collectively influence running biomechanics, load tolerance and RRI in youth runners (Malina, 1994; Lloyd et al., 2014; United States track field, 2020). Notably, early sport specialization in adolescent athletes is associated with increased sport-related injury risk, attributed in part to the homogeneity of movement patterns repetitively stressing the same immature tissues (Hamill et al., 2012; Post et al., 2017). Although associations between running biomechanics and overuse injuries have been widely studied in adults (Ceyssens et al., 2019; Hollander et al., 2021a), comparatively little research has targeted potential relationships between musculoskeletal injury and growth-related changes in biological age, body anthropometry, neuromuscular control, and running biomechanics in youth (Krabak et al., 2020). The aim of this perspective article was to provide a brief summary of the current epidemiology and etiology of RRI. Special consideration is given to the transition in physiological characteristics (such as bone mineral content) that occur around puberty. We subsequently discuss how growth, maturation and sex may all influence running biomechanics, tissue load and RRI risk in adolescent athletes.

## Epidemiology and Etiology of Running-Related Injuries in Adolescents

The growth in participation in youth running has seen a parallel increase in the incidence of RRI in adolescents (Mehl et al., 2011). Prospective studies of high school cross-country runners have reported a wide range in the cumulative seasonal incidence of RRI for girls (34% to 47%, corresponding to 16.7 to 19.6 per 1000 athletic exposures [AEs]) and boys (26% to 48%, corresponding to 0.9 to 15.0 per 1000 AEs) (Beachy et al., 1997; Rauh et al., 2000, 2006). The most commonly injured body locations for high school cross-country runners for both sexes are the shin and knee (Rauh et al., 2000, 2006). Among middle school cross-country runners, the incidence of RRI injuries has been observed at 10.9 per 1000 AEs and 8.0 per 1000 AEs for girls and boys, respectively (Beachy and Rauh, 2014). A cross-sectional study involving 2,113 middle school runners (average age 13.2) found self-reported RRI were more prevalent in girls than boys (56% vs. 50%, *p* = 0.007) (Wu et al., 2021). Girls reported more ankle sprains, patellofemoral pain and shin splints than boys, while boys more frequently reported plantar fasciitis, iliotibial band syndrome and Osgood-Schlatter Disease (Wu et al., 2021) than girls. A separate report in this cohort identified bone stress injury (BSI) was more common in girls than boys (6.7% vs. 3.8%, *p* = 0.004) with the tibia, metatarsus and fibula the most common anatomical locations of injury (Tenforde et al., 2021).

Understanding the etiology, treatment, and prevention of RRI requires identifying associated intrinsic and extrinsic risk factors (Meeuwisse, 1994; Bahr and Holme, 2003; Rauh et al., 2011). A recent consensus statement on youth runners provided a comprehensive evaluation of risk factors and their relationships with musculoskeletal injuries from prospective cohort and retrospective studies (Krabak et al., 2020). Previous injury (Rauh et al., 2000, 2006; Plisky et al., 2007; Reinking et al., 2010; Tenforde et al., 2013; Tirabassi et al., 2016) and sex (female) have been the most consistent intrinsic risk factors for RRI among adolescent runners (Rauh et al., 2000, 2006; Plisky et al., 2007; Tenforde et al., 2013; Tirabassi et al., 2016; Hollander et al., 2021b). Other frequently examined intrinsic risk factors significantly related to RRI include muscle weakness (hip abductor, knee extensors and flexors) (Luedke et al., 2015), Q-angle ≥ 20 degrees (Rauh et al., 2007), increased hip internal rotation range of motion (Yagi et al., 2013), and leg-length inequality greater than 1.5 cm (Rauh, 2018). In female adolescent runners, menstrual dysfunction (Barrack et al., 2014; Rauh et al., 2014) and low bone mineral density (Barrack et al., 2014; Rauh et al., 2014) have been significantly associated with RRI and are the best established risk factors for BSI. Furthermore, it has been shown that risk for BSI is related to the number of accumulated risk factors (Tenforde et al., 2013; Barrack et al., 2014).

To date, there are several extrinsic risk factors that have been identified that contribute to RRI in high school cross-country runners. These include low step rate (Luedke et al., 2016), higher weekly mileage (Tenforde et al., 2011), and infrequently alternating short and long training mileage or running predominantly on hills during summer months (Rauh, 2014). While a recent study indicated that female high school cross-country runners who were classified as high sport specializers were at a two-fold greater risk of musculoskeletal RRI than female high school runners classified as low sport specializers (Rauh et al., 2018), Garcia et al. found no association between sport specialization and RRI among male and female high-and middle-school cross-country runners (Garcia et al., 2021). The differences may be in part due to differing study designs, sample of runners, and injury definitions (Rauh et al., 2018; Garcia et al., 2021).

## Adolescent Running Biomechanics

### Kinetics and Temporospatial Variables

It is generally assumed that the kinetics and temporospatial metrics of adolescents during running are largely similar to adult runners when effects of body height and mass are removed. This assertion, however, has not been tested due to the limited studies describing these metrics in adolescent runners.

As age increases, step length during running increases while step rate decreases (Schepens et al., 1998). The increased step length is attributed to the age-associated increase in leg length (Schepens et al., 1998) as a longer leg length has been associated with lower habitual step rate in adult runners (Tenforde et al., 2019). Prior to the age of 12 years, the decrease in step rate is associated with a decrease in the mass-specific whole body vertical stiffness (ratio of vertical ground reaction force and vertical displacement of the center of mass) due to an increase in body mass with a constant stiffness (Schepens et al., 1998). However, from 12 to 18 years of age, vertical stiffness and step rate are approximately constant due to a parallel increase in both stiffness and mass with age (Schepens et al., 1998).

As running speed increases, not surprisingly, both step rate and length increase, regardless of age. Of note, despite maximum running speed increasing with adolescent age, the relative contributions of step rate and step length to maximum speed change with age or more precisely maturity. In those not yet achieving peak height velocity (an indicator of skeletal maturity), maximum running speed was best predicted by step rate, while step length was the key factor in those post-peak height velocity (Meyers et al., 2017). In adolescents near and above peak height velocity, step rate and horizontal propulsive force during maximum speed running remain relatively constant across age (Schepens et al., 1998; Rumpf et al., 2015). Additionally, greater peak vertical ground reaction force during maximum speed running is evident in older versus younger adolescents even when accounting for the increased body mass (Rumpf et al., 2015). Kinetic and temporospatial measures commonly described in adult runners, such as horizontal braking force, vertical loading rate, and step width, have not been characterized in adolescent distance runners.

### Footstrike Mechanics and Kinematics

Most habitually shod adolescents make initial contact with a rearfoot strike [i.e., initial contact with the heel (Hoenig et al., 2020)] when running at slower speeds (Hollander et al., 2018; Latorre Roman et al., 2019). However, the prevalence of rearfoot strike patterns depend on running speed, the use of and habituation to footwear, age and sex (Hollander et al., 2018). Non-rearfoot strike patterns [i.e., making initial contact with the toes or midfoot (Hoenig et al., 2020)] have been advocated as a potential injury prevention strategy in adolescents and adults (Lieberman et al., 2010), although the evidence is conflicting especially for youth runners (Warr et al., 2015; Davis et al., 2017; Chan et al., 2018; Messier et al., 2018; Anderson et al., 2019; Krabak et al., 2020).

While decreasing loading rate is one arguable benefit of a non-rearfoot strike pattern (Lieberman et al., 2010; Davis et al., 2016), a non-rearfoot strike is not required to achieve low loading rates (Stiffler-Joachim et al., 2019) and the evidence associating high loading rates to RRI development in adults are conflicting (Zadpoor and Nikooyan, 2011; Bredeweg et al., 2013; Kuhman et al., 2016; Davis et al., 2017; Dudley et al., 2017; Messier et al., 2018). Moreover, the results from several studies note that the biomechanical benefit of specific footstrike patterns are conflicting (Stearne et al., 2014; Davis et al., 2016; Dudley et al., 2017), which has been supported by recent prospective injury studies in adults (Warr et al., 2015; Kuhman et al., 2016; Messier et al., 2018; Anderson et al., 2019). Other evidence from studies on adult runners suggest that footstrike more likely affects the risk of specific injuries, rather than one footstrike pattern being more or less injurious than another (Hollander et al., 2021a). Further research encompassing the effect of footstrike pattern on adolescent running gait relating to injury is warranted.

## Implications for Prevention of Running-Related Injuries

### Bone and Bone Mineral Content

Peak bone mass accrual is achieved by early adulthood and is influenced by biomechanical stressors to bone along with other health characteristics (i.e., diet; body weight and lean tissue, and hormonal function). A review of the influence of sports participation on bone density and strength suggests that running is less osteogenic than sports involving higher ground reaction forces in multidirectional loading, such as European football (e.g., soccer) and basketball (Tenforde and Fredericson, 2011). Participation in ball sports for two or more years prior to puberty may promote higher bone content and stiffer, more fracture resistant bones (Milgrom et al., 2000) and reduce future risk for BSI in adolescence and adulthood (Fredericson et al., 2005; Tenforde et al., 2013, 2021).

Adequate bone adaptations according to loading biomechanics require appropriate nutrition, sleep and hormonal function. The Female Athlete Triad and Relative Energy Deficiency in Sport (De Souza et al., 2014; Mountjoy et al., 2018; Tenforde et al., 2020) describe the consequences of low energy availability (defined as inadequate caloric intake vs. energy exercise expenditure) on bone density and hormonal function (including menstrual dysfunction). Caloric needs may be significantly higher during growth and to meet demands of the sport of running (Krabak et al., 2020). In addition to promoting adequate energy availability, ensuring appropriate calcium from food and vitamin D supplementation may promote bone gains and reduce risk for BSI (Kelsey et al., 2007; Sonneville et al., 2012; Barrack et al., 2017). While not adequately studied in adolescent runners, sleep is critical to promote growth and reduce risk for injury and should be duly considered in future research specific to this cohort (Copenhaver and Diamond, 2017).

### Muscle

Lower extremity muscle strength is generally regarded as an important component of RRI prevention programs. The underlying rationale being that muscle weakness leads to altered running mechanics and reduced tolerance to loading, thereby increasing one's risk of common joint and soft tissue injuries. However, lower extremity muscle weakness has not been consistently shown to contribute to injury in the youth runner, similar to findings involving adult runners (Thijs et al., 2011; Mucha et al., 2017). One prospective study's findings of greater injury risk among high school runners with weak hip abductors, knee extensors and knee flexors, Luedke et al. (2015) is contrasted by another study suggesting increased hip abductor strength and hip abduction to adduction strength ratio increases injury risk (Finnoff et al., 2011). Despite this uncertainty regarding muscle weakness and injury risk, injury prevention programs with elements of high intensity neuromuscular training, jumping, plyometrics and balance training have been successful in reducing sports injuries in youth athletes (e.g., basketball, soccer, football, volleyball) (Emery et al., 2005; Rössler et al., 2014; Richmond et al., 2016). Such studies have not been conducted in youth runners, so the effectiveness of these programs remains unknown. Among adult runners, prevention programs emphasizing general lower extremity strength training have not reduced injury incidence (Toresdahl et al., 2020), though a program focused on foot muscle strengthening led to a 2.4-fold lower injury rate (Taddei et al., 2020).

### Tendon

Tendons transfer load between the muscle and bone, are important for efficient movement and provide a mechanical buffer to protect muscle (Roberts and Azizi, 2010; Konow et al., 2012). In running, high-stress tendons, such as the Achilles tendon, store and release energy with each stride like a spring. The better the Achilles tendon functions at this task, the better the economy of running (Alexander, 1990). Tendon dimensions are typically coupled closely to those of the corresponding muscle, and are optimized for efficient muscle contraction and movement (Ker et al., 1988; Alexander, 2002). Both tendon and muscle respond to mechanical loading by becoming larger and stronger (Kubo et al., 2001; Lambertz et al., 2003). In youth, the muscle fascicles and tendon lengthen proportionally during maturation, as does tendon cross-sectional area and the physiological cross-sectional area of muscle (O'Brien et al., 2010). However, the time course of adaptation may be very different (Mersmann et al., 2017), as one study has reported a lag of 1–2 months in tendon property response to resistance training in healthy adults (Kubo et al., 2010). The concern during adolescence is that there seems to be an even greater imbalance in the time course of growth between tendon and muscle properties (Mersmann et al., 2017). Load bearing tendons respond to load by improving their material properties (i.e., elastic modulus; tensile strength) in early adolescence, with hypertrophy (i.e., cross-sectional area change) occurring in the later stages of adolescents (Waugh et al., 2012; Mogi, 2020). The muscle, on the other hand, develops progressively during adolescence and this theoretical lag in tendon adaptation, especially in size, may be the reason for the occurrence of overuse tendinopathy in youth athletes. There is also a mismatch in the growth rate of the muscle-tendon unit relative to that of bone during puberty, in which changes in tendon moment arm do not always remain proportional to the changes in muscle and external moment arm length which can lead to increased load on the tendon with running (O'Brien, 2016). Another concern is that tendon tissue also responds to different kinds of stimulus than muscle. While plyometric activities, such as running and jumping, can result in improved muscle strength, plyometrics do not appear effective in improving tendon stiffness in adults (Bohm et al., 2014). Tendon, on the other hand, seems to preferentially respond to strength training exercises with high loads that strain the tendons over a longer duration than ballistic activities (Arampatzis et al., 2010). If a youth runner does not perform any other type of activity, such as strength training, the tendon might not get the proper stimulus to get larger and stronger, and instead there is a greater risk for developing tendinopathies.

Excessive loading of tendons is considered a risk factor for tendinopathy (Millar et al., 2021). To address the overloading of tendons during running one would have to (1) decrease load through training or technique modifications, or (2) improve the tendon's resistance to load (i.e., stiffness). Compared to adults, children develop lower levels of muscle force at slower rates (Asai and Aoki, 1996; Grosset et al., 2005; Falk et al., 2009; Gillen et al., 2019). Implementing a strength training program for youth runners would address this relative muscle weakness and also improve the tendon's stiffness and, in doing so, improve the rate of force production of the muscle tendon unit (Waugh et al., 2014). This is an area of ongoing research in children.

### Sensorimotor Aspects of Running in Adolescents

The greater vulnerability to foot and ankle injuries of children aged between 10 and 14 years (Lambers et al., 2012; Doherty et al., 2014) is widely related to the challenging phases in motor control development due to the rapidly changing physiological characteristics (e.g., anthropometrics, hormones) (Davies and Rose, 2000). Although most aspects of the association between growth and sensorimotor control are still unknown, there is evidence that basic coordination skills are highly adaptable during childhood and adolescence (Quatman-Yates et al., 2012). This includes phases of quick improvements and steady states, although some individuals may show temporary declines in balance abilities during peak height velocity (Davies and Rose, 2000; John et al., 2019b; Schedler et al., 2019). An indication for the development of sensorimotor control in gait is the long-lasting decline in stride-to-stride variability, which is exceptionally high during the early childhood years and decreases constantly until teenage years (Hausdorff et al., 1999; Petersen et al., 2010; Kraan et al., 2017). A high adaptability of the sensorimotor system to lower extremity balance, strength, power and postural stability (and associated running biomechanics) in the adolescent years is shown in studies using neuromuscular training intervention (Faude et al., 2017; Hopper et al., 2017).

An indication that development of sensorimotor control during childhood may depend on footwear habits is provided by John et al. (2019a). These investigators demonstrated that maturation led to a steady state of balance performance in 11–14 year-old boys who used shoes for sports and recreational activities. Participants of the same age who grew up habitually barefoot, however, continued to increase their balance performance during adolescence (John et al., 2019a). This finding may explain the benefits of habitual barefoot activities regarding a possible reduction of lower limb injury risk in adolescents from Kenya (Aibast et al., 2017). However, the evidence for the influence of footwear on musculoskeletal pain and injuries is limited (Hollander et al., 2017; Francis et al., 2018). Likewise, the evidence regarding the effects of running injury prevention strategies in children or adolescents is scarce. In adult runners, inconsistent findings were reported for the effects of different running shoe properties (insoles, cushioning) (Ryan et al., 2014; Nigg et al., 2015; Hulme et al., 2017). Regardless of the sport, adolescent athletes seem to benefit from neuromuscular training interventions not just for injury prevention but also regarding injurious biomechanics and sensorimotor control (Faude et al., 2017; Hopper et al., 2017). However, future research is needed to confirm or refute these effects in youth running athletes.

## Discussion

It is clear that limited evidence exists on the influence of biomechanics on RRI risk during growth and development in youth runners. Nonetheless participation in running-based activities has significant health benefits. Running is positively associated with improved movement competencies including stability and control skills (Kriemler et al., 2010; Lubans et al., 2010; Krabak et al., 2020). Running can also improve cardiorespiratory fitness while also decreasing the risk of obesity, thereby impacting long-term issues such as heart disease and diabetes (Kriemler et al., 2010; Lubans et al., 2010). Thus, running represents an easy and effective method for youth to meet current exercise guidelines of 60 min or more physical activity per day (Bull et al., 2020).

Unfortunately, there have been no scientific reports to support specific evidence-based training recommendations for youth runners (Krabak et al., 2020; Scheer et al., 2021). Published recommendations regarding appropriate training volumes and distances are mostly based on opinions of healthcare professionals and coaches (Jenny and Armstrong, 2013; Blankson and Brenner, 2016; Finley et al., 2017; Scheer et al., 2021). Youth-specific running programs associated with running clubs (e.g., Boston Athletic Association, Students Run Los Angeles) have attempted to address the risk of injury in these youth running training programs, but more research is needed (Students run LA, 0000; Miller et al., 2018). Despite these limitations, it seems reasonable to suggest that youth participating in a supervised training program are theoretically more likely to be physically and mentally prepared for running events and are potentially at a lower risk for injury and burnout than those youth not participating in supervised training programs (Krabak et al., 2020).

With the goals of reducing overall running-related loads and those specific to the injured tissue, gait retraining is an emerging rehabilitation-specific strategy to modify the biomechanics contributing to injury in the impaired runner. Gait retraining considerations may be deemed appropriate for the injured runner with non-resolving symptoms, following a period of relative rest and reintroduction of gradual increases in running training volume (Davis and Futrell, 2016; Krabak et al., 2016b). If implemented correctly, the demands placed on the injured tissue are reduced thereby promoting recovery and potentially mitigating risk of recurrence. While resistance training increases the load capacity of tissue, the strength gains do not typically improve the running biomechanics associated with injury risk (Willy and Davis, 2011). Several running retraining approaches have been suggested, such as increasing step rate or transitioning to a non-rearfoot strike, and the biomechanical effects of each are well-described in adults (Heiderscheit et al., 2011; Chumanov et al., 2012; Adams et al., 2018; Yong et al., 2018; Napier et al., 2019; Zimmermann and Bakker, 2019). Despite few clinical trials, the findings have been consistently positive in that appropriately applied running retraining improves patient-reported outcomes and injury recovery (Noehren et al., 2011; Willy et al., 2012; Helmhout et al., 2015). For example, increasing step rate or using a non-rearfoot strike improved symptom resolution and return to full running in adults with patellofemoral pain (Roper et al., 2016; Bramah et al., 2019; Dos Santos et al., 2019), likely due to the accompanying reduction in patellofemoral joint loading (Lenhart et al., 2014, 2015). Importantly, running retraining can induce secondary biomechanical changes that must be considered, such as an increase in Achilles tendon loading with non-rearfoot strike (Baggaley et al., 2017). As such, selecting an appropriate running retraining approach should not be viewed as one-size-fits-all but instead be based on the individual's injury characteristics, running mechanics, and running-related goals.

To date, no trials among adolescent runners have been reported using running retraining as part of an injury rehabilitation plan. A recent trial found that healthy adolescent runners did not modify their footstrike pattern after 10-week running retraining programs designed to promote a transition to a non-rearfoot strike (González et al., 2021), suggesting unique approaches may be necessary in this population. Nonetheless, given the increased risk of shin pain in high school runners and of BSI in collegiate runners using a lower step rate (Luedke et al., 2016; Kliethermes et al., 2021), running retraining in this population may be considered as part of a comprehensive treatment plan.

**Figure 1:**
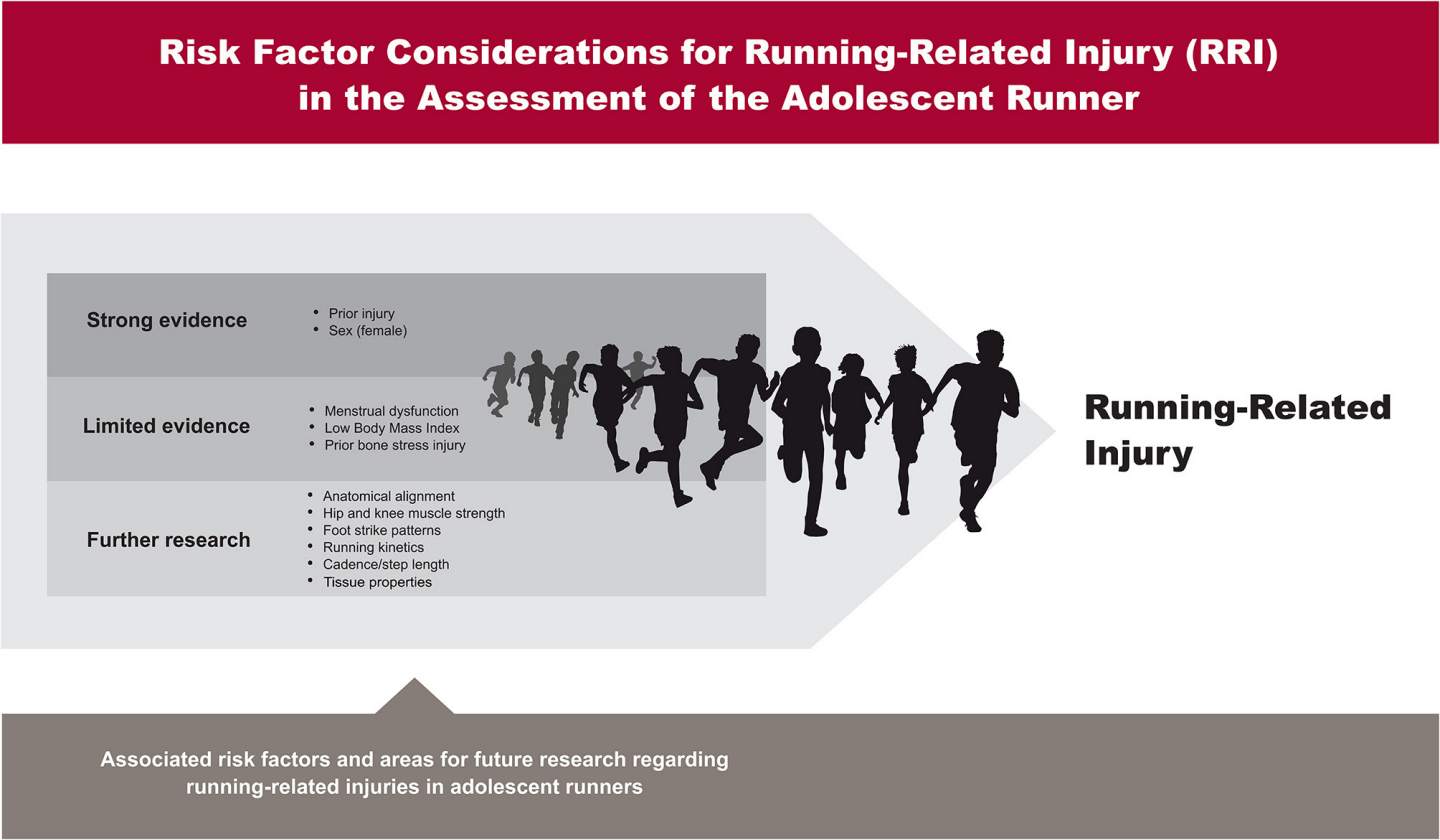
Risk factor considerations for running-related injury (RRI) in the assessment of the adolescent runner.

Based on current evidence, several factors should be addressed in the youth runner with the goal of preventing or rehabilitating injury. There is strong evidence to support prior injury and sex (i.e., girls) as risk factors for future RRI (Krabak et al., 2020), and more limited evidence to support menstrual dysfunction and low BMI to development of stress fractures (Field et al., 2011; Tenforde et al., 2015). Assessments focused on anatomical alignment, strength or flexibility deficits, footstrike kinematics, running kinetics and neuromuscular control need more robust research, but represent potential opportunities for injury prevention. Furthermore, it should be considered that in recent years a more complex model for injury risk has emerged (Bittencourt et al., 2016). This model described by Bittencourt et al. (2016) is certainly to be taken into consideration for assessing injury risk in youth runner, as these non-biomechanical and biomechanical factors may interact with each other. Therefore, athletes should be comprehensively clinically screened for the known factors with further interventions, as appropriate (Figure 1).

This perspective article has provided a summary of the current knowledge and future considerations for research in adolescent running biomechanics and injury prevention. Ultimately what is missing are comprehensive longitudinal studies monitoring changes in running biomechanics and associated musculoskeletal tissue. Such data is critical for the effective design of injury prevention and return to running programs that not only foster optimal performance but also promote healthy musculoskeletal development in adolescents.

## Data Availability Statement

The original contributions presented in the study are included in the article, further inquiries can be directed to the corresponding author.

## Author Contributions

SM, KG, AG, BH, BK, MR, AT, SW, AZ, and KH: substantial contributions to the conception or design of the work, drafting the work or revising it critically for important intellectual content, provide approval for publication of the content, and agree to be accountable for all aspects of the work in ensuring that questions related to the accuracy or integrity of any part of the work are appropriately investigated and resolved. All authors contributed to the article and approved the submitted version.

## Conflict of Interest

The authors declare that the research was conducted in the absence of any commercial or financial relationships that could be construed as a potential conflict of interest.

## Publisher's Note

All claims expressed in this article are solely those of the authors and do not necessarily represent those of their affiliated organizations, or those of the publisher, the editors and the reviewers. Any product that may be evaluated in this article, or claim that may be made by its manufacturer, is not guaranteed or endorsed by the publisher.
